# Raising an Indigenous academic community: a strength-based approach to Indigenous early career mentoring in higher education

**DOI:** 10.1007/s13384-022-00542-3

**Published:** 2022-07-16

**Authors:** Rhonda Povey, Michelle Trudgett, Susan Page, Michelle Lea Locke, Matilda Harry

**Affiliations:** Western Sydney University, Sydney, Australia

**Keywords:** Indigenous, Higher education, Mentoring, Strength based, Culturally responsive, Community cultural wealth

## Abstract

This paper reports on Indigenous early career researchers’ experiences of mentoring in Australian higher education, with data drawn from a longitudinal qualitative study. Interviews were conducted with 30 Indigenous participants. A consistent theme in the findings and contemporary critical literature has been a reaction against institutionalised and hierarchical cloning and investment models of mentoring that reinforce the accumulation of White cultural capital, in favour of strength-based relational models tailored to build Indigenous cultural wealth in parallel with career development. We write from an equity-based standpoint addressing mentoring as a complex and raced space where individual Indigenous ECRs articulate a desire and will to develop a successful and meaningful career, rich in cultural wealth and with their identity intact. It is our intent that these findings will also have global significance and support the more sustainable and ethical career development of First Nation early career academics in relationally like colonised contexts.

A review of literature about mentoring in higher education reveals an iterative research space describing the theory behind and the efficacy of mentoring paradigms and practices (Walters et al., 2016). Awash with the terminology of mentors, supervisors, advisors, tutors and coaches, who support the career development of their mentees, protégées and trainees (Lunsford et al., 2017; Stanley et al., 2005), it is clear mainstream mentoring in the higher education sector is well researched and embedded in institutional discourse. The literature agrees on the importance of mentoring for early career researchers (ECRs),^1^ citing significance for career development and satisfaction, capacity building of job knowledge and research skills (De Janasz et al., 2004; Ewing et al., 2008), socialisation into the workforce, increasing scholarly productivity (Hubball et al., 2010) and academic and research development (Devos, 2008).

Yet for Indigenous ECRs in Australia, mentoring grounded in White hegemony compounds Indigenous educational inequity: Walters et al. (2016) contend a significant factor influencing this disparity in the retention and/or attrition of Indigenous academics is linked to the lack of culturally relevant mentoring. Data from Universities Australia (2020) exposed a disparity between non-Indigenous and Indigenous academic staff, to the extent that an additional 1185 Indigenous academic staff would need to have been employed to attain population parity of 3.1% between Indigenous and non-Indigenous academic staff. We are using the above as a stepping board to engage in constructive and restorative conversations about how to navigate positive and emancipatory transformations in Indigenous higher education. Drawn from an Australian Research Council 3-year longitudinal study *Developing Indigenous Early Career Researchers,* the authors of this paper are promoting a strength-based, culturally relevant approach to Indigenous ECR mentoring. We are investigating Indigenous experiences and perceptions of mentoring, considering the racist precedents and consequences of institutionalised mentoring, and most importantly, listening to Indigenous advocacy for effective mentoring experiences.

We are particularly interested in discovering the impact of Indigenous ways of being, knowing and doing on mentoring practices of Indigenous ECRs in Australian universities and how a strength-based mentoring paradigm might meet the needs of Indigenous academics. This paper reflects a significant shift towards culturally diverse responses to mentoring, collaborative growth and strength-based frameworks, and, in order to move beyond imposed conservative constraints designed to support status quo and ensure longevity of White influence in the sector**,** will address the need for socio-culturally relevant mentoring programmes for Indigenous academics. A study investigating an Australian Indigenous approach to mentoring is wanting, because, despite repeated attempts to improve the models, mentoring paradigms continue to exclude and/or poorly serve the needs of Indigenous peoples in higher education. This is most particularly felt by Indigenous ECRs who are poised on their career trajectory to affect change for Indigenous Peoples (Locke et al., 2021). We also hope that our findings will have global significance and support the more sustainable and ethical career development of First Nation early career academics in relationally like (Goldberg, 2009) colonised contexts.

## Are higher education mentoring models a good fit?

As noted above, literature relating to mentoring in the sector is plentiful. Therefore, what follows is a carefully customised review and commentary on mentoring paradigms, practices and policies that particularly influence the career trajectories and experiences of Indigenous ECRs in Australia. While mentoring is deemed an important foundation for a successful academic career, it is nevertheless facing criticism on many fronts, with literature increasingly contesting the underpinning ideological constructs.

## The institutionalisation of mentoring

Although formal mentoring, built on a dyadic structure of mentor, has been widely promoted (Darwin et al., 2009), its long-standing reputation can be misleading. Scandura (1998) argues that relationships can be complex, and therefore, dyadic mentoring paradigms are fraught with a propensity to become dysfunctional, providing an intractable terrain for the growth of negative, even destructive relationships, as individual and/or institutional needs change. Most important to our intent, formal mentoring programmes and mentoring relationships have been criticised as “mechanisms thorough which relations of power are enacted, maintained and sustained” (Sutherland-Smith et al., 2011, p. 331). Australian universities had previously relied on industry funded research, but a shifting political and economic landscape saw a structural realignment in the sector to funding models dependent upon winning nationally competitive grants and the procurement of grants to validate researcher position as member of an institution (Darwin & Palmer, 2009). This more competitive and neoliberal environment features a corporate ideal of a good researcher, governing performance in accordance with institutional norms measured by performativity (Thunig et al., 2020). Correspondingly, institutions apply greater pressure on their early career academics to perform, conform and maintain status quo. While performativity may be perceived as desirable by institutions, strategies and techniques of formal mentoring can be experienced by Indigenous ECRs as racially biased, marginalising and exclusionary, with an expectation of the academic becoming “governable subjects” (Sutherland-Smith et al., 2011, p. 34), who carry a desire for assimilation and integration into the sector.

## Investment model of mentoring

Embedded within dominant mentoring models is the investment model, best described as a system wherein mentoring is primarily set up for faculty members to meet prescribed re-appointment, promotion and tenure expectations (Dolan et al., 2018). In this way, mentoring represents an investment in human capital of the mentee, ensuring productivity of the faculty member as measured by hard metrics of research and output of publications (Louie, 2019). Designed to integrate the ECRs into the organisational culture, investment mentoring models “replicate androcentric and Eurocentric values that centre individualism” (Endo, 2020, p. 171). Bourdieu et al. (1977) proposed education plays a significant role on an individual’s capacity to accumulate dominant cultural, social and economic capital, creating communities whose “knowledge, skills and abilities are valued by privileged groups in society” (Yosso, 2005, p. 76). Often these investment mentoring initiatives are dependent on the accumulation of the White middle-class social and cultural capital of a sector whose foundations are built on the stolen lands of Indigenous Peoples (Thunig & Jones, 2020) and White constructs of intellectual imperialism (Moreton-Robinson, 2011; Scheurich et al., 1997). Endo (2020) argues mentoring paradigms, such as investment models, are “implicitly designed to assist new employees in understanding the hidden curriculum or the unspoken rules of an organisation’s culture” (p. 171), to ensure productivity; an assurance that the university has made a sound investment in the selecting the early career researcher. It follows that mentoring is problematised for Indigenous ECRs when their career trajectories are shaped and constrained by the hegemonic mentoring relationships and goals. Windchief and Brown (2017) report First Nations academics are excluded from career building interactions with the dominant culture, for example, Women of Colour may be perceived as oppositional, and Lewis (2016) contends Indigenous ECRs may be excluded by gatekeepers in formally produced cultural capital settings, such as being assigned as the primary investigator in research projects, or editors of journals.

The investment model also accounts for the institutional proclivity towards ‘rising star’ phenomena, wherein the pathway to success is illuminated for particular mentees who are the best fit with institutional goals and deemed most likely to succeed, thereby achieving performative success (Devos, 2008). This practice, favourably biased towards those who are compliant and fit the normative expectations, is found to be not only inequitable, but also demoralising for Indigenous early career academics whose academic capital does not grant academic tenure. Additionally, the academic pipeline, built on the premise that the most expedient route to success is linear trajectory (Ysseldyk et al., 2019), does not account for Indigenous ECRs who may take divergent paths, or face discriminatory systemic barriers within the sector (Pihama et al., 2019). Naepi (2019) described the academic pipeline as leaking and pakaru (broken). Investment models of mentoring are antipathetic to diversity: it is clear mentoring frameworks built on White middle-class accumulation of the capital of Western education and language, and social capital of networks and connections, are inherently discriminatory.

## Cloning model

A North American study in 1981 showed mentoring programmes for ECRs were most successful when the career of the protégé becomes a replica of the mentor, hence, the term cloning (Blackburn et al., 1981). In more recent research, Windchief and Brown (2017) studied the intersectionality of race with mentoral cloning, describing the propensity for academic cloning as a “subtle and contemporary assimilation” (p. 336) of Indigenous Peoples. Their research showed the importance of the mentors influence in supporting mentee/protégé to bring White cultural and social capital to the table, thereby creating an insider/outsider relationship that links academic productivity and advancement of protégé. Success is dependent on following the same career path as the mentor. Girves et al. (2005) argue mentoring is much more than “academic advising and role modelling” (p. 453); the reality is best described as grooming of the mentee to become a best fit for the institution. The cost of grooming practices is great, demanding essentialising of the mentee’s cultural identity as well as being positioned to become culturally invisible along the road to success (Walters et al., 2019).

## Critical mentoring paradigms

That dominant mentoring models don’t address the “sociocultural specific challenges and realities” (Endo, 2020, p. 169) of People of Colour who are early career academics, and who must differently navigate the institutional landscape, is self-evident. Hidden in plain sight have been issues of systemic inequity in mentoring policies, characterised by racial neutrality (Alarcón et al., 2017), discrimination and exclusionary practices that disenfranchise those early career academics who may not share the values of economically aligned goals of an enterprise university (Sutherland-Smith et al., 2011), the symbolic violence enacted on those who are unfamiliar with the dominant reproductive codes of culture and power (McDonough et al., 2021) and neoliberal values of individuality and competitiveness (Baice et al., 2021). Indigenous scholars’ determination and audacity has provoked a rejection of these formalistic mentoring models, with much needed and increasing research focussing on relational, inclusive, culturally responsive and diverse paradigms of mentoring practices and policies for Indigenous students and faculty in higher education (Brayboy et al., 2014; Coff et al., 2019; Endo, 2020; Liou et al., 2016; Loban, 2014). Indigenous led critical analysis is increasingly challenging the ideological foundations of institutional mentoring, revealing the negative implications for Indigenous academics, especially for ECRs. In its place, strength-based mentoring models are gaining traction.

Therefore, the question of whose knowledge counts needs to be addressed. Here it serves our purpose well to discuss a particular culturally responsive, strength-based approach. The theory of Community Cultural Wealth (CCW) (Yosso, 2005). CCW is predicated on acknowledgement of the multiple strengths of People of Colour and is grounded in Tribal Critical Race Theory, a theory of significance to People of Colour and Indigenous Peoples. CCW challenges Bourdieuian market-place metaphors of economies of exchange, such as cultural capital, wherein capital is framed as an exploitative power which is accumulated to be exchanged for economic and symbolic profit to gain social and professional advancement (McDonough & Abrica, 2021). Yosso reframes cultural capitals as cultural wealth, aiming to expand what counts as cultural capital and build on CCW. However, as a point of contestation, Neri et al. (2021) argue that Yosso’s use of the term capital to describe the various components of CCW, such as familial capital, aspirational capital and so on, is by default linked to an economics of exchange and so at odds with the notion of a sharable cultural wealth, generating conceptual problems that confound the social justice intent of CCW. This is a fine but important point when discussing Indigenous strength-based mentoring models. Viewed through a critical lens, with Neri et al. (2021), we argue the term *wealth* is better than c*apital* to describe and analyse the multiple cultural resources developed and shared by communities of Indigenous Peoples and People of Colour, but not recognised by institutions.

CCW nevertheless represents and highlights Indigenous cultural resources for empowerment, collective struggles and the Indigenous principles of respect, relationality and reciprocity, stewardship and paying it forward (Brayboy, 2015). Culturally responsive, equity-based approaches, as described above, open up our understanding of the potential for critical strength-based practices and policies to challenge ongoing macro- and micro-level racism and marginalisation of Indigenous and Peoples of Colour (Acevedo et al., 2021) by providing a mechanism to acknowledge what Indigenous ECRs bring to the table, to reveal racial discrimination hindering the building of a productive and meaningful academic careers, and as a guide for non-Indigenous mentors to understand ways to share power and privilege (Stanley & Lincoln, 2005). These policies and practices carry specific relevance to Indigenous ECRs who look for leadership and guidance in the early stages of their careers.

## Methodology and method

### An Indigenist research methodology approach

Indigenist research methodologies underpin this study and refer to a research paradigm that respects and honours Indigenous epistemologies, ontology and axiology. Indigenist paradigms are relational and grounded in the principles of an emancipatory imperative, political integrity and the privileging of Indigenous voices (Rigney, 1999). Smith (1999) contends Indigenous voices must be centred in research, especially given the history of exploitation, prejudice and ‘speaking for’ that has dominated the academic domain. Therefore, throughout this paper Indigenous voices and scholarship are deliberately centred as an agential response to the historical dominance of Eurocentric ideologies in academic discourse (Kovach, 2010). Additionally, the authors of this paper are four Indigenous academics and one non-Indigenous ally.

## Sampling and recruitment

Interviews are being conducted over a 3-year period, with the first round commencing in 2020. This paper reports on this early-stage data of the first round of interviews. Purposeful sampling was conducted (Punch et al., 2014) using a number of different methods. This approach was necessary due to the lack of a publicly accessible document that identified Indigenous Australians with a doctoral degree (Trudgett et al., 2016). Authors two, three and four used professional networks to build a list of potential participants. A social media post on Twitter described the project and provided an email contact for interested Indigenous ECRs, and a database search was conducted on Trove to identify Indigenous theses published between 2015 and 2020. Additionally, a review of previous and current holders of ARC Discovery Grants was compiled. In sum, 58 ECRs were contacted using email addresses sourced through institution directories, LinkedIn, ORCID and Twitter, with 34 accepting, four of whom later withdrew for personal reasons. In response to the small number of Indigenous ECRs in Australia, all participants were deidentified, choosing a pseudonym to avoid identification. Deidentification also supports the moral imperative of the study ‘to do no harm’ and to protect participants’ privacy as well intellectual property rights (Povey et al., 2019).

The demographic distribution of the participants is diverse. The 30 participants represent at least 35 Aboriginal and Torres Strait Islander Nations, employed in 21 universities across Australia at the time of writing and working across a range of faculties (Locke et al., 2021). Although classified as being an ECR, the participants are of different ages, in different stages of their career development, at different academic levels and in different roles. 73% of the total number of participants have been employed in higher education for more than 6 years, a time period longer than the PhD journey. While we offer the findings of this study as snapshots of Indigenous ECR experiences and perceptions, we also propose the ethnographic nature of the qualitative study brings a broader understanding and a much-needed Indigenous perspective on career development in Australian higher education.

## Data collection, coding and analysis

All interviews in the first stage of the study were conducted online and voice recorded using Zoom technology due to the limitations imposed by Covid-19 restrictions in Australia at that time. Despite the necessity of conducting interviews online, using an Aboriginal Yarning method (Bessarab et al., 2010), an Indigenous research method that supports participants cultural safety, helped create a familiar and non-intrusive method. It is also likely participant’s experienced greater cultural safety when being interviewed within the comfort of their own home, rather than in an institutional space. All transcripts were transcribed by a professional transcription process, checked by the fourth author and then emailed to each participant, in accordance with the protocols of ethical research with Indigenous Peoples. Data was initially coded by the fourth author, using Nvivo 12, a qualitative software package that facilitates analysis of data in depth and from a variety of viewpoints (Bazeley, 2013); a key factor especially considering the variations in participants experience working in the sector. Data was first coded according to demographic factors and then thematically. In preparation for thematic analysis, the first author further coded the data by seeking out any crossovers between themes that might be relevant to the mentoring focus of this paper.

## Findings

### Learning the ropes

Navigating the workplace was a key issue raised by participants and deemed to be an area where mentoring was most helpful; this is despite the varied demographic of the participants. As a newly conferred ECR, Sarah sought knowledge through formal mentoring about institutional mechanisms, such as *first time running a grant*, and navigating the system to *learn the institution's rules.* Though more experienced in the sector, Maree also deemed being mentored by an experienced colleague as a key factor in successfully navigating in the sector:

*They're the ones that have been in the game for a long time and used to winning these big grants so learning from them the ropes is paramount, as well.*

Similarly, Patricia identified an ongoing need for support to navigate the system, predominantly through informal mentoring structures:

*So it has been my peers that I’d walk in to their office and go, what do I do now? How do I construct this email?*

For these participants, who came into the ECR role with only some professional knowledge, operational knowledge was a requisite factor for effective mentoring in terms of learning how to navigate the hard metrics of the system. Operational mentoring was not restricted to formal or dyadic mentoring. As an ECR with more experience in the sector, Cate contended:

*I feel like I get mentored by so many other people in - just by observing and watching, as well. That's got to be included as mentorship, because that's their knowledge and their wisdom, their experiences are benefitting me just by being an observer.*

Jessie, reflected on co-writing with experienced colleagues as a highly valued experience:

*How do I take this and turn it into really good journal articles and then where do I send them - Which publishers are going to work best for me. I'm trying to learn that by doing some co-writing on some action research.*

Either through formal mentoring, breaking down the fabrication of power in institutions that were not created with Indigenous academics in mind, or creating supportive collegial relationships, ECRs used initiative and navigational skills to position themselves favourably in the sector.

## Who is holding the ropes?

However, in a sector that is not created by or for Indigenous academics, participants also identified systemic and institutional racism, and a hostile culture in the sector, as problematising mentoring experiences. The competitive culture embedded in the institutional meritocracy is echoed in mentoring relationships between Indigenous mentees and non-Indigenous mentors. Eli explained:

*I think good mentorship is that capacity building, and not being competitive - actually having a genuine investment in the improvement of our experiences within this white institution.*

Eli continued, describing the institutional culture as *toxic,* in its *competitiveness*. This toxicity is commonly associated with politics of the institution grounded on meritocracy and individual success; *the ways it is set up*. Moreover, toxicity is also attributed to racial discrimination. Stopping short of calling it lateral violence, Wiraga’s story tells of the ominous presence of unequal power relations. They identify key challenges as:

*Negotiating the bureaucracy and the positioning of [tribal affiliation] perspectives against [...] how that can actually manifest. You're always having to watch your back. It's the politics of place.*

James’ concerns also centred on hidden agendas, and hierarchical structures, proposing:

*I think mentorship needs to avoid as much as possible […] hierarchy […] So, you go seeking mentorship, but it's not always to your benefit. It's usually to build - because you are, within a white institution, and you're surrounded by non-Indigenous Peoples. You're actually building their profile, rather than building your own*

Participants were forthright in calling for culturally relevant and responsive mentoring that addressed the particularities of an Indigenous ECR in a Eurocentric institution. A paucity of Indigenous academics to fill the role of mentors then is of great concern: Lee explained why this matters:

*I would really like an Aboriginal mentor, because there’s just stuff that I have to encounter that other people don’t encounter. Trying to get around it is really challenging.*

Olive, who had not received any form of formal mentoring, bemoaned the *lack on the ground of Indigenous mentors.* Additionally, Olive was disappointed about the paucity of Indigenous mentors for research *with time to actually help me with anything.* Participants also propose cultural awareness was absent from their experiences of mentoring, with Olive arguing: *How do I make this argument that Aboriginal people think [it] is important?*

## Taking the reins

Despite extensive experience in higher education, Julunybarr understood the specific needs of Indigenous academics who are building careers. They explained the significance of an Indigenous mentor who has a shared history and a shared understanding of the place of culturally congruent relationships in mentoring. In particular, Julunybarr spoke about the importance of caring and nurturing, of spiritual health:

*So, I think if we're mentoring Aboriginal and Torres Strait Islander, we need to have a little bit more understanding of the triggers that might be there for them. I think we also need to know how to advocate on behalf of them and who to go to if it's outside our expertise. I think we do need to have downtime to be able to be just people with each other, you know, and look after each other. I think that's really important because I think our mental health is just as important as well as our spiritual health - to think about how to get things done on time, the timeline stuff.*

Skywalker also addressed relationships:

*Fundamentally it’s about a relationship There’s a reciprocal nature to it which implies the whole thing around authority and power as well and I think there’s got to be some – there’s got to be a de-centring of that somewhere in that relationship for me [...] the relationship is the key and especially with our mob there’s shared contexts and understandings, and things like that that differentiate the relationships that I have, mentoring relationships that I have with Aboriginal people than I do with non-Aboriginal people.*

Similarly**,** Wirraga placed high value on the place of Indigenous perspectives on the sector and the passing on of this knowledge between mentor and mentee:

*I think it's helping, it's the tricks along the way of interpretation, interpretation of the bureaucracy that is the university and it's also through a cultural lens, through engaging in Indigenous space*.

Shaun and Areau presented a familial understanding of mentoring, one that acknowledges Indigenous ways of intergenerational knowledge transmission and the place of community and Elders in knowledge production. Shaun stated

*But I've been blessed in other ways through relationships and community members. There's a number of community members that were basically my support system. Most of my mentoring's probably in my community. There's a couple of aunties and a couple of uncles and brothers that I get to talk to a lot.*

Areau’s contribution also acknowledged the significance of community in supporting an academic career, clearly articulating why Indigenous mentoring matters:

*The academy don't understand community in the same kind of way so they don’t see it as that constant teaching and learning that our community mentors, that our community engages with, that our Elders expect of us that we would pass on our knowledge*.

Sometimes grown from frustration and disappointment, yet always driven by a determination to shape positive change, the 13 participants cited in this paper have, in the spirit of collegiality and trust, shared their perceptions and personal experiences of mentoring. As advocates for Indigenous equity in education, these proud Indigenous ECRs are shining the light on improved and just mentoring practices that are equity-based, collaborative, non-hierarchical and relational.

## Discussion: best-fit approaches to Indigenous ECR higher education mentoring

Findings from this study, as voiced by the 30 Indigenous ECR participants, confirm the criticisms made by critical, Indigenous and critical race theory scholars of mainstream mentoring. Calling for culturally responsive equity-based approaches, Indigenous ECRs are challenging the ahistoricism that decontextualised mentoring, advocating for the acknowledgement of Indigenous epistemologies, axiology and ontology within the sector (Martin, 2003). Participants in this study have very clearly described what can be learnt from their experiences of ECR mentoring across 21Australian universities. We have learnt from our participants about the perpetuation of inequity and racial prejudice that underpins formal mentoring models: findings show dissatisfaction with formal mentoring programmes, especially those that neglect to place an “emphasis on social, historical and political contexts which shape our (Indigenous) experiences, lives, positions and futures” (Martin, 2003, p. 205), and so fail to support the growth of Indigenous cultural wealth in a system founded on Western epistemologies.

The following discussion is grounded in acknowledgement of individual and cultural uniqueness. We do not propose a singular approach as we accept the inherent caveat that as the backgrounds, academic trajectories and individual needs of Indigenous mentees vary, so will mentoring needs also vary. This position also acknowledges the limitations of a one-size-fits-all model of institutionalised hierarchical mentoring structures (Alarcón & Bettez, 2017). Rather, we write from an equity-based standpoint (Endo, 2020), addressing mentoring as a complex and raced space where individual Indigenous ECRs articulate a desire and will to develop a successful and meaningful career, rich in cultural wealth and with their identity intact (Walters et al., 2019).

Formal mentoring is one such approach seen as potentially problematic by participants, a position confirmed by scholars critical of investment and cloning models of mentoring (Blackburn et al., 1981; Dolan et al., 2018; Sutherland-Smith et al., 2011). Girves et al. (2005) advise formal mentoring is most effective when embedded in university infrastructures, encouraged by university leaders and aligned with university goals of recruitment and retention. Accordingly, several participants agree that formal mentoring supports early career academics navigate the system, through granting access to the expertise and knowledge of the mechanisms driving the system. Passing on the accumulated knowledge of navigating this environment, whether this be about writing grants, preparing journal papers for publication, or discerning the appropriate wording of emails, was highly valued in terms of learning the tricks of the trade. Yet although this position is deemed to be successful in contexts where the mentees’ values, beliefs and positionality are in accord with their university, when it comes to navigating “adverse environments” (Windchief & Brown, 2017, p. 333), several participants spoke of resistance to these extant mentoring models, in particular when colonial governance predicated on a cultural bias assumes Indigenous academics “must change to fit in and conform to what is deemed as an already effective and equitable model” (Yosso, 2005, p. 75).

In place of hierarchical models that sustain racial injustice and lack of parity in higher education, Indigenous ECRs champion non-hierarchical models that are collaborative, include multiple mentoring relationships that align with Indigenous ways of being, knowing and doing (Martin, 2003), and culturally safe and informed cross-race mentoring relationships (Stanley & Lincoln, 2005). In particular, participants advocate for best practice mentoring that promotes culturally congruent models, thereby supporting Indigenous ECRs’ capacity to build and share wealth that is interconnected and relational: participants argue effective mentoring requires a sense of connectedness between the mentor and mentee, a position described by Loban (2014) as a way of “knowing, navigating, surviving and succeeding in the universities’ tricky terrain” (p. 14). Participants also advocated for mentoring guided by Indigenous relationality, of experiencing the self as part of others, learnt through “reciprocity, obligation, shared experiences, coexistence, cooperation and social memory” (Moreton-Robinson, 2020, p. 16). For example, Indigenous scholar Karen Martin’s Quandamooka ontology, grounded in Indigenous ways of being, knowing and doing, is a good fit with the mentoring paradigms detailed in this study. In line with the position taken by Chew et al. (2021), participants agreed relationality enhances lasting and meaningful mentoring relationships.

Participants confirmed equity-based literature and Indigenous academic leaders by promoting mentoring built on and calling upon community knowledge. Loban (2014) contends good mentoring entails passing on this accumulated wisdom and is quick to point out that this wisdom goes beyond knowledge about and held within the walls of the institution, a concept framed by Brayboy (2013) as nation building: mentoring built on community knowledges that carries a legacy of service to past and future generations. Similarly, familial cultural wealth paradigms posit empowering Indigenous mentors and mentees to use “assets already abundant in community” (Yosso, 2005, p. 82). This perspective reinforces the importance of seeking guidance from Indigenous community within the institution and from family, Elders and community outside the institutional walls. As we have seen, Indigenous ECRs come into the field from diverse demographics, bringing with them traditional epistemologies, ontology and axiology to engage in a multidimensional academic journey, and not necessarily with a linear career trajectory. Indigenous ECRs contend they are well placed to continue to draw on community for guidance and are actively promoting the integration of community into their mentoring experiences.

In sum, Indigenous ECRs are telling of resistance to an imposed view, questioning who is holding the ropes and expressing a desire for Indigenous academics, family and community to be involved in holding reins by using “our own processes to articulate our experiences, realities and understandings” (Martin, 2003, p. 211). Drawing on Indigenous cultural wealth positions Indigenous ECRs to bring strength-based approaches with them to their academic role and act with autonomy from a position of Indigenous sovereignty. Windchief & Brown’s summation (2017) is poignant, proposing that mentoring is constructed with respect for cultural integrity, relevance, reciprocity, and responsibility, so mentees have an “opportunity to contribute in a meaningful way and build capacity with Indigenous identities strengthened through validation of lived experiences” (p. 33).

## Conclusion

### Raising an Indigenous academic community

Findings in this study show unambiguous calls for Indigeneity to sit at the heart of equity-centred mentoring paradigms for Indigenous ECRs. A consistent theme in the findings and contemporary critical literature has been a reaction against institutionalised and hierarchical cloning and investment models of mentoring that reinforce the accumulation of White cultural capital, in favour of strength-based relational models tailored to build Indigenous cultural wealth in parallel with career development. As described by the participants, Indigenous mentors are highly sought after and valued by Indigenous ECRs, who seek equity and acknowledgement that what they bring with them to the institution can be used to benefit and protect the richness of unique cultural identities. With a desire to build on and extend critical mentoring practices in the Australian context to incorporate Indigenous ways of knowing, being and doing, participants in this study are aspirational in the intent to build a robust Indigenous academic community.

To conclude and complete the circle, we return to the beginning of our paper with a reminder of the disparity in the number of Indigenous academics in Australian universities, an issue of concern because fewer Indigenous academics in the sector means fewer Indigenous academics available for mentoring Indigenous ECRs. As a closing comment, we propose because the disparity in Indigenous academics impacts negatively on ECR career trajectory, the core business of universities must be to support increased numbers and retention of Indigenous academics with secured funding for Indigenous strategic plans, by embedding Indigenous leadership in the sector (Trudgett et al., 2021) and by endorsing strength-based cultural wealth mentoring. As argued by Endo (2020), equity-centric mentoring in higher education is well positioned to support the retention of Indigenous academics beyond recruitment. Creating parity by building Indigenous presence in the sector and developing culturally safe and community-connected universities for Indigenous Peoples is a necessary pre-requisite for the decolonisation of higher education.

## Footnotes

1 The term ECR is favoured by the Australian Research Council to describe academics within five years of post-doctoral confirmation.
